# The impact of migration on the sexual health, behaviours and attitudes of Central and East European gay/bisexual men in London

**DOI:** 10.1080/13557858.2013.789829

**Published:** 2013-04-19

**Authors:** Richard C.M. Mole, Violetta Parutis, Christopher J. Gerry, Fiona M. Burns

**Affiliations:** a School of Slavonic and East European Studies, London, UK; b Research Department of Infection and Population Health, Centre for Sexual Health and HIV Research, University College London, London, UK; c Royal Free Hampstead NHS Trust, London, UK; d National Centre for Social Research, London, UK

**Keywords:** sexual health, migration, homosexuality, sexual behaviour, sexual attitudes, Central and Eastern Europe, London

## Abstract

**Background:**

Building on an earlier quantitative study which found that gay/bisexual men from Central and Eastern Europe were at greater risk of sexual ill health following migration to the UK, the aim of this qualitative study is to explore how the process of migration itself may have influenced the migrants’ sexual behaviour and attitudes.

**Methods:**

To address these questions, we conducted 17 in-depth interviews in London with gay/bisexual male migrants from Central and Eastern Europe, drawing on Fisher and Fisher's Information-Motivation-Behavioral Skills model as an interpretive framework.

**Results:**

We find that the sexual behaviours of our respondents have been significantly influenced by the process of migration itself. In particular, extricating themselves from the traditional systems of social control in their home societies and having greater access to gay venues in London resulted in their increased sexual activity, particularly in the first phase of migration. High-risk sexual behaviour was found to be a factor of sexual mixing, the use of commercial sex and perceptions of risk in the UKvis-á-vis Central and Eastern Europe, with each of these factors also influenced by the process of migration. Risk-prevention behaviour depended upon the possession of appropriate risk-prevention information, motivation to use condoms and appropriate behavioural skills, with the latter two factors in particular influenced by social mores in the home country and the UK.

**Conclusions:**

The interviews suggested a number of migration-related factors that increased the STI and HIV risk for these migrants. A number of potentially important policy recommendations stem from our analysis.

## Introduction

The accession of 10 former communist states to the European Union in 2004/2007 led to the migration of hundreds of thousands of Central and East Europeans to the UK. While, according to the theoretical literature, most will have been driven by the desire to boost their social capital and earning potential, for one group of migrants – lesbian, gay, bisexual and trans (LGBT) migrants – factors other than higher wages and work experience may have played a greater role in their decision to migrate (Stark and Bloom 1985). While the legal situation for LGBT people in Central and Eastern Europe (CEE) has improved since the collapse of communism, with homosexuality subsequently decriminalised in a number of states, social attitudes to non-hetero-sexuality remain largely negative and the ability to live openly without fear of persecution or violence is more restricted than in many Western states (Amnesty International 2008).^1^ Migration, therefore, offers LGBT individuals from CEE ‘a means of escape and of self-realisation’ as much as economic betterment (Binnie 1997, 240). Anecdotal evidence suggests that many CEE LGBT individuals have used the opportunity provided by EU Enlargement to move to more gay-friendly cities in Western Europe, with London a popular destination (Graham 2007).

The issues surrounding migration by LGBT individuals have become the subject of a small but growing literature, with a primary focus on domestic rural-to-urban migration (Gorman-Murray 2007); LGBT migrants’ motivation to move abroad – primarily in the US context – and the legal hurdles they have to overcome to do so (Cantu 2009); and the construction and reconstruction of their sexual identities following migration (Kuntsman 2009). Our paper aims to broaden the scope of research in this field by analysing the sexual health, behaviours and attitudes of LGBT migrants from CEE. While there exist previous studies on the sexual health of LGBT migrants in the Chinese and US contexts (He et al. 2007; Somerville et al. 2006), ours is the first broad-based analysis of the sexual health, behaviours and attitudes of CEE LGBT migrants in the UK.

## CEE LGBT migrants in the UK

Most CEE migrants arriving in the UK are young, single and without dependants, factors suggesting that they are likely, or wanting, to be sexually active.^2^ Yet, as the 1990s witnessed huge increases in rates of HIV and other sexually transmitted infections (STIs) across CEE and as sex education is very limited in the region, gay migrants from the CEE accession states constitute a population at risk of sexual ill health (Waugh 1999, 72–73). To our knowledge, published sources of information on the sexual health of and uptake of safer sex measures by gay/bisexual migrants from CEE are limited to our own recent quantitative work (Burns et al. 2011; Evans et al. 2011a). That study, based upon a large cross-sectional survey, found that gay/bisexual migrants from CEE were:

at significant risk for the acquisition and transmission of STI and HIV through UAI [unprotected anal intercourse] with non-concordant casual partners, sexual mixing and commercial sex. They were more likely to report STI diagnoses when they had been in the UK for longer and a high proportion of STI and HIV were reported as diagnosed in the UK. These findings suggest that CEE MSM [men who have sex with men] are at greater risk following migration to the UK. (Evans et al. 2011a, 328)^3^

However, quantitative approaches are unable to show how the process of migration itself may have influenced the migrants’ sexual behaviour and attitudes. We, therefore, carried out 17 in-depth interviews with gay and bisexual migrants, to examine how their sexual behaviours and attitudes were shaped by the migratory experience.

## Methods

The qualitative research on which this paper is based is drawn from a larger project on the sexual attitudes and lifestyles of CEE migrants in London, which examined the sexual health of heterosexual men and women as well as gay/bisexual men from the 10 CEE accession states. A detailed methodological description has been published (Evans et al. 2011b) and a summary is set out below. The study was approved by the Camden and Islington Community Research Ethics Committee (07/H0722/110).

### Participants

Eligible respondents were literate men aged 18 years or over who self-identified as migrants from one of the 10 CEE accession states.^4^ The sample was recruited from community venues and two sexual health clinics in London between 1 July 2008 and 31 March 2009. To identify sufficient numbers of gay/bisexual men, participants were also recruited in gay venues and the questionnaire was posted on two dating websites: Gaydar and Gay Romeo.

For the purpose of this analysis, i.e. to explore the attitudes and behaviours of men who have sex with men, we treat gay and bisexual respondents as a single group. All respondents who completed the survey were asked if they would participate in an exploratory, face-to-face, semi-structured, in-depth interview. Out of 565 gay/bisexual respondents who completed the survey only 7 agreed to give an in-depth interview. The original study design included recruiting a robust sample of gay/bisexual men using respondent-driven sampling. This would potentially have resulted in larger quantitative and qualitative samples of this population. However, this sampling method failed due to the lack of networking among CEE gay/bisexual men in London (Evans et al. 2011b). However, placing a link to the survey on two gay websites resulted in further 691 respondents, 10 of whom agreed to an interview. In total, 17 interviews were conducted with gay/bisexual men. Interview participants were offered a £15 high street voucher as an incentive. The interviews took place in a university office. Purposive sampling was employed for the interviews to ensure diversity in country of origin, age and time in the UK, although the qualitative sample does not claim to be representative.

### Measures

The interviews were conducted in English or one of the CEE languages depending on the interviewee's preference. All interviews were recorded and transcribed verbatim and translated into English with full adherence to participant confidentiality. Data analysis was facilitated by the use of Atlas.ti software. The analysis used the Framework approach (Ritchie and Spencer 1994), whereby the verbatim data are ordered and synthesised within a thematic matrix, which emerged from both reviewing extant literature and the interview data itself. Pseudonyms have been used to protect participants’ identities.

### In-depth interviews

The main aim of this study was to explore the extent to which the sexual behaviour and understanding of risk of CEE gay/bisexual men in London were conditioned by the migration process. We examined the impact on the frequency of sexual activity of the extrication from traditional systems of social control, feelings of loneliness in the host society and increased access to gay venues in the migration context. To understand risk behaviours, we analysed the impact of sexual mixing, the use of commercial sex, perceptions of risk in the UK vis-à-vis CEE, motivation to use condoms, behavioural skills and possession of appropriate information.

In terms of risk-preventative behaviour, we analysed our respondents’ actions and beliefs with reference to Fisher and Fisher's Information-Motivation-Behavioral Skills model, which maintains that individuals minimise risk only if they possess the necessary information, the motivation to change their risk behaviour and the behavioural skills required to do so (1992, 464). Recognising that in previous studies the relationship between knowledge and safe-sex behaviour among gay men had been equivocal, Fisher and Fisher argued that information on HIV/AIDS was effective only if it is related to preventative behaviour, rather than knowledge about AIDS in general. With information generally understood to be a necessary but not a sufficient condition for AIDS prevention, they argue that ‘even a well-informed and behaviorally skilled person must be highly motivated to initiate and maintain AIDS-preventative behavior,’ with motivation largely influenced by broader social norms (1992, 466). The third of the three conditions, behavioural skills, cover ‘the ability to communicate with, and to be appropriately assertive with, a potential sexual partner’ and ‘the ability to avoid drinking or drug use before sex’ (1992, 468).

In analysing our respondents’ risk-prevention behaviour within this interpretive analytical framework, we argue that the possession of correct information may be incomplete as a result of the lack of sex education in CEE and that our respondents’ motivation and behavioural skills will continue to be influenced by the traditional systems of social control of their home societies – especially in the initial period.

## Results

The sample comprised 2 bisexual and 15 gay men from 7 CEE states, although, in line with general patterns of migration from this region, most were from Poland and Lithuania. The participants were relatively young: the majority (*n* = 14) were aged 25–34 and only three were aged 35–40. Around half reported being in a relationship at the time of the interview. They were relatively recent arrivals: 15 had lived in the UK for up to 4 years with a maximum stay of 12 years in one case. They were well educated: the majority (*n* = 11) had a university degree and the rest had completed at least secondary education.

## Factors increasing frequency of sexual activity

The desire to escape repressive social mores in one's home country and live more openly as an LGBT person is a key factor in the migration decisions of many sexual dissidents (Cantu 2009). The difficult situation for gay/bisexual men in their home societies was the most frequently cited reason given by respondents for wanting to move to the UK. While ‘homosexuality, like all forms of sexuality, has different meanings in different cultures,’ it is, nevertheless, possible to discern some comparable sexual norms and attitudes towards homosexuality and sex education in CEE as a result of their shared communist experience and of the impact of the collapse of state socialism (Weeks 1992, xi).

During the communist period, everyone was expected to adhere to the ‘psychology of the collective,’ with the result that alternative sexualities were considered unacceptable. Homosexuality was further seen as being contrary to the public good as it could not produce children and was thus considered a ‘dangerous sign of individualism’ (Attwood 1996, 102). Communist regimes were hostile to sexuality in general as they sought ‘to ensure absolute control over the personality’ by attempting ‘to deindividualise it, to destroy its independence and its emotional world’ (Kon 1999, 208).

The collapse of state socialism had a massive physical and psychological impact on the local population. Nowhere was this felt more acutely than in the area of reproduction, with the result that fertility rates plummeted. In response to the prospect of their nations ‘dying out,’ politicians and church leaders sought to manipulate fertility rates by ‘working symbolically to delegitimise abortion and empirically to cripple the work of newly established family planning organisations and sex educators’ and by demonising sexual practices that failed to produce children, with homosexuality a favourite target (Rivkin-Fish 2006, 152). The perceived threat of ‘national decline’ thus contributed towards conservative attitudes to sexuality, in general, and negative attitudes to sex education and LGBT rights, in particular, as attested by the repeated attacks on Gay Pride events and violence against gay men and women in CEE over the past decade (Amnesty International 2008).

Many of our respondents had experienced hostility or violence at home as a result of negative attitudes towards homosexuality. As two Polish respondents recalled, being openly gay translates in the minds of many Poles as being mentally ill or a paedophile. Even if one managed to avoid outright hostility, to live as a gay/bisexual man in Poland often meant living a life underground. Positive representations of homosexuality were largely absent in the media. Our respondents commented that, in seeking to keep a ‘normal’ image of themselves and avoid arousing suspicion in their societies of origin, gay/bisexual men in CEE often tried to adhere to traditional gender norms and often got married and had children. In Poland, for example, Abramowicz (2007) found that 80% of gay/bisexual people conceal their sexual orientation in the workplace, 66% hide it within their living environment and 50% conceal it even while living with a same-sex partner. The lack of tolerance of homosexuality in their home societies was also cited by respondents from Lithuania, Hungary, Romania, Bulgaria and Estonia.

For our respondents, the process of moving to London and extricating themselves from the systems of social control of their home societies had an immediate impact on their sexual behaviour. In many cases, migration to the UK resulted in an increased sexual activity, which was especially apparent in the initial period of their stay. Some interviewees did have male sex partners before they emigrated but these relationships were usually limited, constrained by homophobic social attitudes fuelled by the pronouncements of the Catholic and Orthodox Churches, the influence of which grew markedly after the collapse of communism. A few of our respondents had their first homosexual sex after coming to London. For example, Lubor (a 35-year-old Slovak) explained that he was 33 when he first came to London and met his first male partner online: ‘It was so late because I was very internally “paralysed” in my brain by the influence of the Church.’ While many respondents appreciated the fact that homosexuality was discussed openly in British society, some nevertheless felt that, for them, ‘sexuality is too private to talk about’ (Bartosz, 35-year-old Pole). This suggests that the impact of the traditional systems of social control of their home societies and the silence surrounding sex in CEE continued to exert an influence on some of our respondents even after they moved to London.

While Muñoz-Laboy, Hirsch, and Quispe-Lazaro (2009) identified loneliness as a factor for increasing sexual activity among male migrants in the USA, this was not raised as an issue by the respondents in our study. This could be explained by the fact that our respondents are legal migrants – unlike many Mexican migrant men in the USA – and can, therefore, travel back and forth between the UK and CEE freely and inexpensively. This also presents a potential risk factor in itself in that STIs contracted in the UK could be spread to CEE by migrants having sex with partners back home.

Moving from a traditional environment to a city with ‘an explicit “gay scene’” (Chapman et al. 2009, 689) often allows migrants to explore their sexuality for the first time, ‘maximizing their independence and sexual freedom;’ in this context, a ‘clinical or risk-oriented approach to sex would reduce their ability to enjoy these freedoms’ (Chapman et al. 2009, 699). After migrating, many of the men interviewed did indeed try to maximise their sexual freedom and ‘make up for lost time’ by engaging in casual sex with multiple partners. After living in a homophobic society, ‘satisfying your desires was a priority,’ argued Tomas, a 28-year-old Lithuanian, referring to his initial period in the UK.

Living in London also increased access to venues where one could meet other gay/bisexual men. In their home countries, it was the Internet that played the key role in meeting sexual partners due to the fact that there were few gay bars, clubs or other venues. In London, however, our respondents discovered a range of places where they could meet potential partners face-to-face: saunas, cruising areas, gay clubs, pubs and bars, all of which were, according to one interviewee, ‘strictly about sex.’ Adapting to the sexual mores of the London gay scene, respondents realised it was acceptable to engage in sex purely for the sake of sex, without emotions or commitments. As Bartosz, a 35-year-old Pole, described it:

I've been to the gay sauna, which is the place where you go in, pay the money, £15, you leave and you never see those people again…. So I never looked at sex – before London – just as a way to relax. In many cases I tend to treat sex in London as that. There is the point that stress at work and you just want to relax, and you go there and that's it.

## Factors increasing risk behaviour

To better understand risk behaviour in our interviews we focused on sexual mixing, use of commercial sex, perception of HIV and STI risk in the home country vis-à-vis the UK, motivation to use condoms, behavioural skills and possession of safe-sex information. In terms of risk-preventative behaviour, we again draw on Fisher and Fisher's Information-Motivation-Behavioral Skills model (1992).

Recognising that there is a higher prevalence of STI/HIV among certain groups and that sexual mixing can heighten risk by providing bridges ‘for the spread of sexually transmitted diseases from members of one ethnic group to another,’ we sought to understand the extent to which our respondents had sex with men from ethnic groups other than their own (Gras et al. 1999, 1961). Our quantitative study found that compared to British nationals fewer CEE migrants had ever been diagnosed with a STI, suggesting a higher prevalence of these infections in the UK than for the majority of migrants surveyed; this was particularly true of HIV in gay/bisexual men despite similar testing rates (Evans et al. 2011a). Sexual mixing with British gay men thus increased the probability of exposure to an individual with HIV compared to sexual mixing with other CEE men. A number of our respondents, like Bartosz (a 35-year-old Pole), found themselves popular with UK men. Compared with many gay/bisexual men in London, who, some respondents felt, tended to ‘exaggerate their gayness,’ East Europeans ‘are tall… we behave like men, we don't behave like queens. In our society we had to pretend to be straight so we come here and this is the way we are.’ The choice of words in this excerpt is revealing, particularly the slippage from ‘had to pretend to be’ to ‘this is the way we are,’ which becomes especially interesting in the context of discussions of social hierarchies within the gay/bisexual community. Such hierarchies are based not just on perceived masculinity but also racial characteristics and the intersection between the two, as we can see below.^5^

Sexual mixing was evident from most respondents. Many appreciated being able to meet men of various ethnicities – with other European, Latin American and black men mentioned particularly often – as sex partners, although a number of respondents did express a preference for men of their own national group when it came to forming a more lasting relationship, because of the shared language and culture. Stanislaw (a 30-year-old Pole) was insistent on not forming a relationship with a British man because of the potential power disparity. Being with another migrant was considered to be easier because in a Polish-British couple ‘the Pole is in a lower position,’ i.e. the British partner is a full citizen of the state, is fluent in the national language, is accorded a higher status than an immigrant by many co-members of British society and, therefore, more likely to be deferred to in social and financial situations. Many of the respondents felt that East Asian men were too feminine and that Asian men in general, but particularly Muslim men, were too different culturally.

Several men reported selling sex as a source of income. One man worked in escorting before moving to London and continued this line of work after migration. Although not his main source of income, Andor, a 23-year-old Hungarian, admitted that ‘now, since the credit crunch, I do it quite often.’ Another man started to work in this area having found himself in financial difficulties, unable to cope with the high cost of living in London. As previous research on gay migrant sex workers has shown, the potential for increased risk relates to the fact that financial insecurity of the kind experienced by the latter respondent can result in sex workers feeling ‘disempowered to insist on condom use,’ especially if he hopes ‘to lure the client on another occasion’ (Chapman et al. 2009, 699).

While a small number of interviewees did admit to paying for sex, most reported that they did not feel the need to do so while they were still young and attractive, and able to find sex partners. As Roman (a 25-year-old Pole) points out: ‘I'm 25 and why should I pay for something that I can have for free?’ This perception of the motivations for paying for sex chimes with previous research on the customers of male prostitutes, who are characterised as ‘stereotypically inadequate, unattractive, unhappy individuals with no other sexual outlets’ (Morse et al. 1992, 349).

Most interviewees believed that the risk of becoming infected with HIV was higher in the UK than in their home countries. Reasons for this included higher prevalence of HIV, large migration flows, greater ethnic diversity and increased sexually activity. When asked to evaluate the degree of risk associated with their own sexual activity, however, many interviewees said confidently that they considered themselves to be engaging in low-risk behaviour. Rather than risk perceptions being based around sexual behaviour and condom use, however, the former were often formulated by the characteristics of their sexual partners. African partners were viewed as higher risk partners because of the high levels of HIV in Africa. Many interviewees reported that they assessed how ‘risky’ a prospective partner was by his looks. A common phrase used in the interviews was ‘you can usually tell [if the man is healthy]’ (Boyan, a 23-year-old Bulgarian). An association between physical and sexual cleanliness (i.e. presence or lack of infections) was not uncommon. Other men assessed a partner's potential risk by his willingness to use a condom. If he refused or was ‘not keen on using a condom’ (Iulian, a 24-year-old Romanian), they viewed this as a sign of his carrying a high risk of HIV or STI. Some interviewees, like Kazik, a 26-year-old Pole, suggested an association between a partner who had a good supply of condoms and high-risk sexual behaviour:

If I go to have sex at someone's place and I can see dozens of condoms and lubricants in his drawer, I know that sex with him is not safe. This is my feeling because I see and I know that some people jump from one flower to another.

## Risk-prevention behaviour

As most interviewees claimed that they were aware of the risk of STI (including HIV) acquisition, yet considered themselves to be ‘low risk,’ strategies for reducing risk were explored to determine whether their behaviour justified this perception.

Fisher and Fisher argue that, in general, ‘information is a necessary but often not a sufficient condition for AIDS-risk behavior change’ except in those cases where ‘risk-reduction behavior requires a relatively uncomplicated behavioral performance (e.g. avoiding sexual contact, as opposed to acquiring, discussing, and consistently using condoms)’ (1992, 465). In this connection, information would be a sufficient condition for just one of our respondents, Istvan, a 35-year-old Hungarian, who largely abstained from sexual activity altogether due to his fear of becoming infected:

What happens with me in these situations, when I try to get to sex then I keep thinking all the time that I put myself in a very dangerous situation and this destroys everything. This results in having a sexual life close to zero here in London, which is not good, because this is also part of life … This is something that gives me a very hard time.

In cases where the respondents appeared to have a sound understanding of safe-sex practices, the relationship between information and risk-reduction behaviour was ambiguous. More worryingly, it appears as if some of the safe-sex information held by our respondents – e.g. that there is a link between physical and sexual cleanliness – was misconstrued. Other misconceptions expressed by our respondents – for instance, that HIV is curable (Matas, 40-year-old Lithuanian) – demonstrate that not all of our respondents meet the first of Fisher and Fisher's three conditions. Indeed, some of the respondents felt that they were being bombarded with too much information about safe sex and the manageability of HIV, which resulted in their feeling blase and no longer afraid of risk.

Even for the well informed, Fisher and Fisher argue that a ‘person must be highly motivated to initiate and maintain AIDS-preventative behavior,’ with motivation largely influenced by broader social norms (1992, 466). As with all social phenomena, social norms – socially accepted forms of behaviour – are culturally and historically contingent. Social norms governing the use of condoms or the acceptability of sex education in the UK or the Netherlands, for example, will be quite different from those in Poland or Romania.

With regard to the impact of socially embedded norms on risk-reduction behaviour, a recent study on condom use by young gay/bisexual men in the Netherlands (Franssens, Hospers, and Kok 2009) confirmed that the generally ‘favorable attitudes towards condom use’ were a factor of high intention to use condoms, with intention itself a factor of personal norms (11). As all the respondents in this study had a Dutch cultural background, one can assume that the respondents’ personal norms were influenced to some extent by the social norms of the Netherlands, a very liberal state with regard to acceptance of homosexuality and one in which sex education is widespread and the use of condoms encouraged. By contrast, attitudes towards condom use – in a similar study by Kocken, van Dorst, and Schaalma (2006, 235–236) – were found to be far less positive among immigrants in the Netherlands, i.e. among those who did not receive sex education in the Netherlands and who will have been less influenced by Dutch social norms. The authors of the study explained the lower intention to use condoms on the part of immigrants from the Dutch Antilles with reference to Antillean culture, the taboo against discussing sexuality and the reluctance on the part of parents to educate their children about sex. These findings are also relevant for our study. All of our respondents grew up in CEE, where sex education was all but non-existent, homosexual behaviour largely went against social norms and where condom use among men having sex with men has been found to be low (Amirkhanian 2012). As migrants, they did not receive sex education in the UK and will not have been influenced by British social norms – at least not in the initial period of migration. ‘British norms’ are not, of course, shared by all of the UK population, and attitudes towards sex and sexuality are very heterogeneous. Nevertheless, research conducted by Eurobarometer (2006) does show that support for LGBT rights in the UK is higher than in all bar one of the CEE countries. As our respondents are all gay/bisexual and live in London, it is largely the specific norms of the London gay scene that will influence their behaviour.

The influence of social norms regarding condom use may help us understand why UAI among our respondents decreased the longer they were in the UK. The evidence from our quantitative survey suggests that condom use increases with time spent in the UK, even after controlling for increasing sexual activity and commercial sex interactions: among those sexually active in the last 4 weeks, condom use increased from 42% for those in the UK for less than 1 year to over 60% for those present for 2–4 years. This figure then stabilises at just below 50% for those in the UK longer than 4 years. Those who did not use condoms were usually well aware of the risk in which they were engaging and thus undertook frequent sexual health check-ups. This was not perceived to reduce the risk but they believed that it was ‘better to know earlier.’ These examples suggest that even respondents who possess information about risk-prevention behaviour are not always motivated to act accordingly or did not possess the behavioural skills to do so.

The third of Fisher and Fisher's conditions, behavioural skills, cover ‘the ability to communicate with, and to be appropriately assertive with, a potential sexual partner’ and ‘the ability to avoid drinking or drug use before sex’ (1992, 468). The lack of the latter behavioural skill was suggested by the high level of drink and drug use identified in the quantitative arm of the study and further substantiated by Eimis, a 25-year-old Lithuanian, who recounted:

When you meet someone in a club, you have the opportunity to formulate an opinion of the person, you have the opportunity to talk to them and then you can decide. In any case, the decision is often influenced by alcohol. Then you can't really say this is 100% what I would go for if I were sober.

In addition, as discussed above with reference to the lingering effects of the traditional systems of social control and the silence around sex in CEE, not all our respondents were comfortable speaking openly about sexuality. Some interviewees reported that they found it difficult to insist on the use of condoms for fear of losing their partner. Others ignored the risk associated with unprotected sex when they hoped that the sexual encounter ‘may turn into something more’ (Maciek, a 33-year-old Pole). Over time, however, respondents did report becoming more confident in their sexual identity, and this sometimes translated into their being more prepared to take steps to reduce risk, by insisting on the use of condoms, for instance. Some believed confidence helped them find a balance between having an active sex life and engaging in high-risk behaviour, as described by Bartosz:

It's very hard to meet Polish guys who are balanced in the middle, maybe they are in a relationship or open relationship, but if they have partners, they have safe sex and they are confident about themselves, they don't hide from the world, they are confident that they are gay and they are happy about this.

In other words, once they became more confident in their sexual identity to the extent that they felt comfortable living openly in a same-sex relationship, their number of sex partners reduced and with it the risk of infection.

## Conclusions

Our findings provide compelling evidence that the sexual behaviour of gay CEE men has been influenced by the process of migration. Extricating themselves from traditional systems of social control in their home societies allowed the gay CEE migrants in London to engage in casual sex with a large number of partners, facilitated by the more relaxed attitude towards sex and by the greater number of venues where one could meet other gay men and engage in sex. This high-risk behaviour was particularly noticeable in the initial period after migration, when differences in the sexual mores of the two societies were most readily apparent. However, it is not always possible for individuals to escape the cultural and social norms of the homeland.

From the data we presented, it could be concluded that the factors increasing sexual risk for the CEE gay men are: the removal of hostility and violence; widespread liberal attitudes; less imposed religious observance or intolerance; the relief of a ‘paralysis;’ greater opportunities to meet sexual partners and satisfy one's desires. Although all of these factors are normally deemed to be positives in life, in this context they seem to be consistent with heightened sexual health risks.

Our interviews confirm the degree of sexual mixing by CEE gay men identified by Evans et al. (2011b) and help explain why certain ethnicities are preferred over others, with cultural and religious similarities to and differences from the pre-migration society being the main factors. Sexual mixing was also facilitated by a preference for CEE men by some British gay men, which, according to our respondents, was related to the former's perception of masculinity/femininity. While a number of respondents working as escorts in London had also done so prior to migrating, others had moved into this line of work as a result of moving to the UK.

The interviews suggest the information possessed by migrant gay men on risk prevention was frequently misguided. Risk perceptions were often evaluated in the context of certain types of sex partners, sexual satisfaction and substance use rather than sexual practice. Condom use was even viewed by some as a marker of a high-risk sexual partner. Depending upon their individual perception of risk, interviewees employed various risk-reduction strategies, the most popular of which was ‘trying to use a condom.’ In the initial period of migration, the motivation and behavioural skills required to reduce risk were often lacking, the result of social norms brought with them from their home societies, with many men preferring regular check-ups rather than focusing on preventive strategies. Over time, however, as the influence of the social norms of the home society weakened and those of the host society strengthened, the percentage of men engaging in UAI declined and condom use increased. Fisher and Fisher's model enabled us to evaluate the particular importance of motivation and behavioural skills in reducing risk and the impact thereon of social norms. Our data also suggest a rather surprising impact of the widely available sexual health information on gay men's sexual attitudes. Interviewees started to underestimate their vulnerability and the influence that STIs and HIV might have on their health. They developed a range of misconceptions about risk, such as beliefs that HIV can be cured, that a partner's HIV status can be deduced from his appearance or that HIV is only associated with certain types of partners.

## Limitations

The qualitative methodology used in this study limits the ability to generalise these results to all gay CEE migrants in London, although utilising purposive sampling did help ensure diversity in key variables. Moreover, it is not possible to treat a ‘CEE migrant’ as a stable category given the significant differences, among other things, in language, culture and religion between Poles and Bulgarians or Lithuanians and Romanians. Country-specific analysis remains a task for future research. Nevertheless, no other study has generated data on the sexual health, behaviour and attitudes of gay/bisexual CEE migrants in London. Important and nuanced implications for public health policy stem from the analysis.

## Policy, practice and research

These findings highlight that the risk behaviours and knowledge of gay CEE migrants place them at risk of acquiring and transmitting STIs (including HIV). In particular, through interaction with multiple partners from diverse backgrounds, engagement in paid sex and through the acquisition of misinformation regarding sexual risk and treatment, gay/bisexual CEE men are a vulnerable sexual health population. It is, therefore, important that culturally and linguistically appropriate prevention programmes target new arrivals to the UK, in particular, and that the beliefs, attitudes and misconceptions that influence awareness of HIV risk among these men are addressed. More focus is required on the value of preventative (‘safer’) behaviour, which is often viewed as secondary to regular testing for STIs/HIV and is characteristic of the approach to public health adopted in CEE. Protecting and improving the health of these migrant communities will benefit the health of the wider community, both in the UK and CEE.

## Key messages

(1) The sexual behaviour of gay/bisexual CEE migrants in London was significantly influenced by the migration process itself.
(2) It granted men access to a wider range of gay venues and potential sexual partners, increasing their sexual activity, and resulted in greater sexual mixing and the use of commercial sex, both factors in high-risk behaviour.
(3) In the initial period of migration, the motivation and behavioural skills required to reduce risk were often lacking, the result of social norms brought with them from home.
(4) Over time, the influence of UK social norms strengthened and respondents were more motivated to use condoms.

## Notes

1. It should be noted that homosexuality was decriminalised before the collapse of communism in Bulgaria (1968), Czechoslovakia (1962), Hungary (1962), Poland (1932) and Slovenia (1977).

2. Of the CEE migrants who registered with the Workers Registration Scheme between May 2004 and June 2006, 92% was aged 18–44 (Home Office 2006).

3. As lesbians constitute a low-risk group with reference to STIs, they are excluded from this analysis.

4. The sample was restricted to nationals of the 10 CEE accession states as they are entitled to NHS services and the UK government has a responsibility to ensure their health needs are being met.

5. The extremely complex relationship between nationality and sexuality is the subject of much academic interest (see Mosse 1985; Pryke 1998). There is, of course, no *a priori* relationship between nationality and sexuality. While never fully hegemonic and always contested, specific discourses on nationality and sexuality are produced to create moral leadership and social hierarchy and legitimate particular truth regimes.
